# The novel BRDT inhibitor NHWD870 shows potential as a male contraceptive in mice: A novel inhibition of BRDT for male contraception

**DOI:** 10.3724/abbs.2022135

**Published:** 2022-09-30

**Authors:** Sixian Wu, Xiaoliang Li, Lijun Shang, Lvying Wu, Tongtong Li, Peiyv Li, Zhiliang Ji, Jianwen Hou, Mingzhu Yin, Wenming Xu

**Affiliations:** 1 Joint Laboratory of Reproductive Medicine SCU-CUHK Key Laboratory of Obstetric Gynaecologic and Paediatric Diseases and Birth Defects of Ministry of Education West China Second University Hospital Sichuan University Chengdu 610041 China; 2 Reproductive Medical Centre West China Second University Hospital Sichuan University Chengdu 610041 China; 3 School of Human Sciences London Metropolitan University London N7 8BD UK; 4 State Key Laboratory of Cellular Stress Biology School of Life Sciences Xiamen University Xiamen 361102 China; 5 Department of Dermatology Hunan Engineering Research Center of Skin Heath and Disease Xiangya Hospital Central South University Changsha 410008 China

**Keywords:** male contraception, NHWD870, BRDT, BET family inhibitor, spermatogenesis

## Abstract

Small molecule inhibitors of the bromodomain and extraterminal domain (BET) family proteins have emerged as promising options not only for the treatment of multiple cancers but also for disturbing the process of sperm maturation with potential for use as viable contraceptive targets. In this study, we find that the BET family inhibitor NHWD870 and BRDT can bind well *in vitro* through bioinformatics software prediction and protein binding inhibition experiments. NHWD870 can produce a good contraceptive effect through animal experiments *in vivo*, and the fertility can be restored to normal after drug withdrawal. Transcriptomics and proteomics results suggest that NHWD870 affects pathways related to spermatogenesis and maturation, further contributing to the male infertility phenotype. Our results show that NHWD870 can induce a complete and reversible contraceptive effect in mice, which is stronger than that of JQ1 and its synthesized derivatives. This study is expected to eventually lead to clinical trials.

## Introduction

Male contraceptives can be divided into hormonal contraceptives [ 1– 8] and non-hormonal contraceptives [9]. Hormonal contraceptives have potential side effects [ 10– 12] ; despite this, several hormonal agents have entered clinical trials [13]. Non-hormonal contraceptives are at an earlier stage of development [14]; their main targets include retinoic acid receptors [ 15– 17] , testis-specific protein kinases [ 18, 19] , bromine domain protein BRDT [ 20, 21] , ion channels [22], and sperm surface antigens [23].

Bromodomain testis-specific protein (BRDT) belongs to the BET protein family, which includes transcriptional regulators of gene expression through recognition of the lysine acetylation of histone H4 [24]. The BET family includes BRD2, BRD3, BRD4, and BRDT [21], and they have two conserved bromo domains BD1 and BD2 and a terminal domain ET, which mediates protein interactions [25]. A previous study showed that homozygous mice with selective deletion of the BD1 domain present abnormal sperm morphology and that homozygous BD1-deficient mice lose fertility [26], indicating a critical role of the BD1 domain in spermatogenesis.

The *BRDT* gene is specifically expressed in testicular tissues such as pachytene spermatocytes, spermatocytes and round spermatids, and after sperm maturation the expression is mainly located in the nucleus [27]. It plays an important role in identifying H4 histone acetylation in the nucleus of spermatocytes and early round sperm cells [24]. BRDT and BRD4 also contain a C-terminal motif, CTM [25], which is responsible for facilitating the recruitment of other transcriptional regulators ( Supplementary Figure S1A). It was demonstrated that mature sperms or spermatids were absent in the testes of mice with complete BRDT deletion [28]. Furthermore, a genome-wide association study on idiopathic male infertility revealed that the single nucleotide polymorphism of *BRDT* is significantly associated with oligospermia or azoospermia [ 29, 30] . All these studies suggest that BRDT may be considered a target for contraceptive drugs.

In 2010, Matzuk *et al*. [31] studied the effect of JQ1 on the murine reproductive system. Treatment of mice with JQ1 produced a complete and reversible contraceptive effect through its highly specific inhibitory effects on BRDT at the spermatocyte and round spermatid stages [31]. That study established a new contraceptive that can cross the blood–testis barrier and inhibit bromodomain activity during spermatogenesis, providing a lead compound targeting the male germ cell for contraception [31]. However, the relatively high concentration of JQ1 used for an *in vivo* contraceptive effect hinders its potential clinical use, and further investigations are needed regarding its effects for lower concentrations and shorter convalescence.

Recently, JQ1 and different synthesized derivatives based on its structure have been used to target oncogenic properties of the BET family, including GSK525762 (I-BET762), OTX-015, and CPI-0610 [ 32– 34] . BMS-986158 is another drug developed for this purpose but has a different core structure from JQ1 and can bind extremely well with the bromine domain of the BET family [35]. We recently optimized the structure of BMS-986158 and obtained a new drug named NHWD870 [36]. NHWD870 has improved efficacy and pharmacokinetic properties. Our pharmacodynamics results showed that NHWD-870 is three-fold stronger than BMS-986158, 50-fold stronger than JQ1, and 14-fold stronger than GSK525762 or OTX015, indicating that NHWD870 is a very effective inhibitor of the BET family. Consequently, it has been used for suppressing cancer cell-macrophage interactions, among other functions [36].

In this study, we aimed to explore whether our newly developed BRDT inhibitor, NHWD870, could be used as a potential compound for male contraceptives owing to the abovementioned reasons and its improved properties. First, we carefully explored its binding capability with BRDT using molecular docking and protein affinity tests. Then, we checked its testicular bioavailability, spermatogenic effects, and potential pharmacological mechanism through *in vivo* mouse experiments and transcriptome and proteomics analyses. Based on the results, we discuss the potential of developing NHWD870 as a new generation of compounds for contraceptive drugs.

## Materials and Methods

### NHWD870

NHWD870, a small-molecule bromodomain inhibitor of the BET family, is also called pyrido[2′,3′:4,5]pyrrolo[3,2-f]indazole,7-(1,4-dimethyl-1H-1,2,3-triazol-5-yl)-1,9-dihydro-1-methyl-9-[(S)-phenyl(tetrahydro-2H-pyran-4-yl)methyl]. NHWD870 used in this study was prepared and produced as previously described ( Supplementary Figure S1B) [36]. The properties and verification of NHWD870 can also be found in our previous work [36].

### Protein sequence alignment and AUTODOCK fitting

The sequences of BRD4 and BRDT of humans and mice from the UniProt database were used to analyse and predict sequence similarity and conservation. The PDB data of BRDT were downloaded, and AUTODOCK software was used to perform the molecular interaction analysis with NHWD870.

### Protein binding inhibition test

Ten gradient concentrations of NHWD870 were used for single-well detection. AZD5153 was used as a positive control for double-well detection. NHWD870 and AZD5153 were diluted to the final solution with 1000 times dilution in a 384-well source plate. Then, Echo550 was used to transfer 20 nL to a 384-well reaction plate to be tested. In the negative control, 20 nL of 100% DMSO was transferred, and in the positive control, 20 nL of the highest concentration of positive compound was transferred. 5 μL of 4×protein solutions [BRD2(D1, D2); BRD3(D1, D2); BRD4(D1,D2); BRD4(D1); BRD4(D2); BRDT(D1)] was added in each well, followed by centrifugation at 150 *g* for 1 min and incubation at room temperature (RT) for 15 min. Aliquots of 5 μL of 4× peptide solution were added to each well of the reaction plate, followed by centrifugation at 150 *g* for 1 min. After addition of 10 μL detection solution, the 384-well source plate was centrifuged at 150 *g* for 60 s, shaken gently to mix, and incubated at RT for 60 min. EnVision multimode plate reader was used to read the value of each well, and the inhibition rate was calculated using the following formula:

**Table:** 

$$\begin{array}{lr} Inhibition\,\;\;rate\,\;\;\;(\%)\;=\frac{signal\,\;\;max-signal\,\;\;sample}{signal\,\;\;max-signal\,\;\;min}\;\times 100\;\% & \end{array}$$

The dose-response curve was fitted by taking the log value of the concentration as the X-axis and the inhibition rate as the Y-axis. The analysis software GraphPad Prism 5 was used to fit the dose-response curve to obtain the IC _50_ value of the compound in protein binding inhibition.

### Cell culture

GC1 and GC2 cells were obtained from Prof. Jinpeng Sun of Shandong University (Jinan, China) and cultured in DMEM (Gibco, Carlsbad, USA) supplemented with 10% fetal bovine serum (FBS) containing 1% penicillin and streptomycin. TM3 and TM4 cells provided by Guangzhou Women and Children’s Medical Center (Guangzhou, China) were cultured in F-12K (Gibco) supplemented with 5% horse serum and 10% FBS containing 1% penicillin and streptomycin. All cells were cultured at 37°C in the presence of 5% CO _2_.

### CCK-8 assay

GC1, GC2, TM3, and TM4 cells were treated with NHWD870 at different concentrations of 0, 1, 10, 100, or 1000 nM for 0, 24, 48, 72, and 96 h, and their proliferation was assessed using the Cell Counting Kit-8 (Dojindo, Rockville, USA), and the absorbance were measured on a Thermo Scientific Microplate Reader (ThermoFisher, Waltham, USA). All experiments were repeated three times.

### Apoptosis assay

Cells were cultured in 6-well cell culture plates and then treated with NHWD870 at different concentrations of 0, 10, and 100 nM for 12 h. Cells were washed twice with ice-cold 1×PBS, trypsinized, and stained using FITC-annexin V Apoptosis Detection Kit (BD Biosciences, San Diego, USA) according to the manufacturer’s protocol. Stained cells were submitted for analysis on a FACS AriaII flow cytometer (BD Biosciences). All experiments were repeated three times.

### RT-qPCR

Total RNA was extracted from mouse tissues or cells using TRIzol reagent (Invitrogen) and converted to cDNA using a RevertAid First-Strand cDNA Synthesis Kit (ThermoFisher). Real-time PCR was performed using SYBR Premix Ex Taq II (TaKaRa, Dalian, China) on an iCycler RT-PCR Detection System (Bio-Rad Laboratories, Hercules, USA). The ΔΔCT method was used for data analysis. Each assay was performed in triplicate for each sample. The *actin* gene was used as an internal control. The primers for real-time PCR are listed in Supplementary Table S1.

### Animals, testes, and sperm testing

C57 mice (SPF grade) were purchased from Chengdu Dossy Experimental Animals Co. Ltd. (Chengdu, China). The animal experiment was approved by the Animal Ethics Committee of West China Second Hospital of Sichuan University (2021006). All experiments conformed to all relevant regulatory standards. Male mice (6 weeks, 20± 2 g) were divided into five experimental groups according to the different NHWD870 treatments of 0 (control), 1, 2, 3, and 4 mg/kg. Each group contained 10 mice.

NHWD870 was given by intragastric administration once a day for 5 consecutive days, followed by a 2-day discontinuation as a cycle for 3 weeks. Body weights were recorded during this period. Seven of the mice were euthanized 3 weeks after the administration of NHWD870, and the other three mice were subjected to the subsequent mating experiments to observe their recovery after the termination of the drug. One testis from each mouse was weighed, and one epididymis was shredded in PBS. After the epididymis was incubated at 37°C for 30 min to free the sperm, the sperm parameters were analysed using a CASA instrument (Chengdu Puhua Technology Co., Ltd, Chengdu, China). The testis and epididymis on the contralateral side were fixed with 10% formaldehyde for sample fixation and sectioning.

One week after the administration of NHWD870, each male mouse was introduced to two female mice for mating; drug administration was continued for another 2 weeks. After 3 weeks of drug administration, the male and two female mice continued to mate. The timing of pregnancy and the number of litters produced by female mice in each group were monitored and recorded for another 8 weeks.

### Serum sex hormone level determination

Blood samples (0.5 mL) were taken from the eyes of the mice and transferred into a 1.5-mL EP tubes without anticoagulant. After 20 min, the blood samples were centrifuged at 1500 *g* for 20 min. Levels of testosterone, follicle-stimulating hormone (FSH) and luteotropic hormone (LH) in the upper serum were detected by radioimmunoassay using the corresponding test kits ( Shanghai Xin Fan Biological Technology CO., LTD, Shanghai, China ) according to the standard protocol. Finally, the radioactivity count of the sex hormones in the animal serum samples was determined with a gamma counter (XH6080; China National Nuclear Corporation Xi’an Nuclear Instrument Factory, Xian, China). According to the standard curve, the serum testosterone, FSH, and LH hormone levels of each sample were calculated.

### Rat animal experiment

Nine male SD rats (200 ±10 g, 8 weeks; SPF grade) were purchased from Chengdu Dossy Experimental Animals Co. Ltd. (Chengdu, China), and they were evenly divided into three groups. NHWD870 was administered to rats by the following three routes: 1 mg/kg by tail vein injection, 4 mg/kg by gavage, and 4 mg/kg subcutaneously for 3 weeks using the same protocol as in the mouse experiments. The liver, testis, and epididymis of the rats were collected for sectioning and H&E staining.

### H&E staining

The testes of mice or rats were fixed in 4% PFA for 30 min at RT. Fixed testes were embedded in paraffin, and 5-μm-thick sections were prepared. These sections were deparaffinized, and H&E staining was performed according to standard protocols.

### Immunohistochemical microscopy

After antigen thermal restoration on the rehydrated paraffin sections, the sections were immersed in hot 0.01 M sodium citrate solution and then cooled naturally to RT. After cooling, the sections were transferred to PBS for 5 min to remove the endogenous peroxidase activity. The paraffin sections were rinsed with distilled water and then soaked in PBS containing 0.3% Triton X-100 for 20 min. The sample section was circled with an immunohistochemical pen and blocked with 1–2 drops of 5% normal goat serum (diluted in PBS) for 15 min. After removal of the blocking solution, the sections were incubated with primary antibody overnight at 4°C. The sections were then washed three times (5 min each) with PBS, followed by incubation with the HRP-conjugated secondary antibody. Finally, DAB developer was used to develop color, and photos were taken and examined under a light microscope (Olympus, Tokyo, Japan).

### Immunofluorescence assay

After antigen retrieval on paraffin, sections of mouse testis were fixed with 10% formaldehyde, and incubated with the primary antibodies including anti-Ddx4 (1:500; Abcam, Cambridge, UK), anti-Plzf (1:100; Abclonal, Wuhan, China), anti-Pcna (1:100; Abclonal), anti-Ki67 (1:500; Abcam), and anti-Cyp11a1 (1:50; Abcam) overnight at 4°C. Then, fluoresceine-lablled secondary antibodies (1:1000; Bio-Rad, diluted with 1% BSA-PBS) was added and incubated for 1 h at RT in the dark. After three times (5 min each) wash with PBS, the slide was mounted with a mounting tablet containing DAPI. Photos were taken under a fluorescence microscope (Olympus) for observation.

### Transmission electron microscopy

The testis tissue was prefixed with 3% glutaraldehyde first, postfixed in 1% osmium tetroxide, dehydrated in acetone, infiltrated in Epox 812 and embedded. The semithin sections were stained with methylene blue, and ultrathin sections were cut with a diamond knife and stained with uranyl acetate and lead citrate. Sections were examined with a JEM-1400-FLASH transmission electron microscope (JEOL Ltd., Tokyo, Japan).

### Transcriptomics

To define the effect of NHWD870, transcriptomic analysis was performed on male mice treated intragastrically with a 4 mg/kg dose for 3 weeks. The testes of three mice were collected for transcriptomics analysis, while the testes of three untreated mice were used as a control group.

Total RNA was extracted from testicular tissues and the integrity of the testicular RNA samples was detected with an Agilent 2100 Bioanalyzer (Agilent, Santa Clara, USA). A NEBNext® UltraTM RNA library building kit (New England Biolabs, Ipswich, USA) was used to synthesize the cDNA libraries, and an Agilent 2100 Bioanalyzer (Agilent) and RT-PCR were used to identify the quality of the cDNA library. The qualified cDNA library samples were sequenced at both ends by Illumina sequencing. Clean read data were then evaluated by Q20, Q30, and GC content calculations and mapped to the mouse reference genome (GRCm39) using HISAT2 v2.2.1 [37]. Annotated reference genes were identified using the mouse genome GTF annotation file (GRCm39.104) by StringTie v2.1.5 [38]. Differentially expressed genes at the transcription level were examined using the R package DESeq2 (1.32.0) [39].

### Proteomics

In parallel with the transcriptomics analysis, a proteomics analysis was also performed. After total testicular proteins were extracted, the testicular protein peptides were desalted and freeze-dried in vacuo with Strata X C18 (Phenomenex, Torrance, USA). Using Strata X C18 (Phenomenex). After the freeze-dried peptides were dissolved in 0.5 M TEAB, the peptides of the testis protein samples were labelled according to the operating instructions of the TMT kit (ThermoFisher). The peptides of the testicular protein samples after TMT labelling were fractionated by high-pH reversed-phase HPLC using an Agilent 300Extend C18 column with the following parameters: 5 μm particle size, 4.6 mm inner diameter, and 250 mm length. The peptides were fractionated using pH 9, 8%–32% gradient acetonitrile, and 60 components were separated within 1 h. Then, the 60 separated peptide components were combined into nine components. The combined protein fraction of the testis from the NHWD870-treated group and the control group were vacuum freeze-dried and then subjected to subsequent analysis.

The testicular protein sample peptides were dissolved in mobile phase A of liquid chromatography, separated by the EASY-nLC 1200 ultrahigh-performance liquid system, and then entered into the NSI ion source for ionization analysis. The obtained secondary mass spectrometry data of testis protein samples in the NHWD870 group and the control group were retrieved by Maxquant 1.5.2.8 [40], setting the TMT-6plex as a quantitative method and the FDR to 1%. Quantification of proteins and analysis of differentially expressed proteins were carried out using the R package Proteus [41].

### Western blot analysis

Proteins of the mouse testicular tissues were extracted using a universal protein extraction lysis buffer (Bioteke, Winooski, USA) containing a protease inhibitor cocktail (Roche, Basel, Switzerland). The denatured proteins were separated on 10% SDS-polyacrylamide gels and transferred to a polyvinylidene difluoride (PVDF) membrane (Millipore, Billerica, USA). Then, the membrane was blocked for 0.5 h at room temperature and incubated overnight at 4°C with the following primary antibodies: anti-Cep112 (1:1000; ThermoFisher), anti-Na/K-ATPase (1:500; Abclonal), anti-Spata6 (1:1000; ThermoFisher), anti-Wdr60 (1:500; ThermoFisher), anti-Akap3 (1:1000; Abcam), and anti-Tubulin (1:500; Abclonal) antibodies. After being washed with 0.3% TBST, the membrane was incubated with the horseradish peroxidase-conjugated secondary antibodies (1:5000; Abclonal) for 1 h at room temperature. Then the blots were visualized with the gel imaging system (Beijing Liuyi Biotechnology, Beijing, China).

### Bioinformatics analysis

Simulation experiments on the binding force between NHWD870 and the BET family were carried out with Gromacs 2020.4 [42] using the Amber99SB force field [43] and the TIP3P model of water. Homology modelling was constructed using the protein structure data of the BET family from the PDB Database. The similarity of the homologous template was above 0.75.

### Statistical analysis

All data are expressed as the mean±standard error of the mean (SEM). All statistical analyses were performed using GraphPad Prism software (v8.00). To compare differences between the two groups, the *t* test was used. One-way analysis of variance (ANOVA) was used for statistical comparison of the above two groups. For the comparison of multiple groups, we used one-way ANOVA and then used Dunnett’s *t* test and SNK-q to make a pairwise comparison after obtaining the difference. *P*<0.05 was considered statistically significant.

## Results

### Molecular interaction between NHWD870 and BRDT

**Figure 1:**
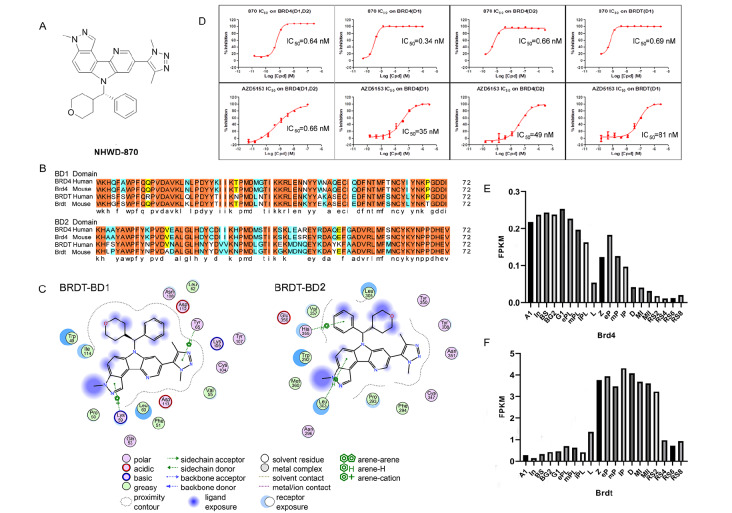
Figure 1 Structure simulation and protein binding assays show strong binding of NHWD870 to the BD1 domain of BRDT (A) The molecular structure of NHWD870. (B) Alignment results of human and murine BRD4 and BRDT BD1 and BD2 domains. (C) Molecular interaction between BDRT and NHWD870. The interaction mainly involves the connection between aromatic rings and hydrogen, as well as the interaction between aromatic rings and cations. (D) The IC 50 values of protein binding inhibition. AZD5153 was used as a positive control. The IC 50 values of NHWD870 on BRD4 (BD1, BD2), BRD4 (BD1), and BRD4 (BD2) were 0.64 nM, 0.34 nM, and 0.66 nM, respectively, while the IC 50 value of NHWD870 on BRDT (BD1) was 0.69 nM. (E,F) The mRNA expression of Brd4 and Brdt in spermatogenic cells at various stages in mouse testes. Single cells were selected from GEO. In total, 20 subtypes of spermatogenic cells were profiled, including differentiated spermatogonia (A1, type A1 spermatogonia; In, intermediate spermatogonia; BS, S phase type B spermatogonia; BG2, G2/M phase type B spermatogonia), preleptotene spermatocytes (G1, G1 phase preleptotene; ePL, early S phase preleptotene; mPL, middle S phase preleptotene; lPL, late S phase preleptotene), meiotic cells (L, leptotene; Z, zygotene; eP, early pachytene; mP, middle pachytene; lP, late pachytene; D, diplotene; MI, metaphase I; MII, metaphase II), and round spermatids (RS2, step 1–2 spermatids; RS4, step 3–4 spermatids; RS6, step 5–6 spermatids; RS8, step 7–8 spermatids).

The molecular structure of NHWD870 is shown in Figure 1A. Initially, we compared the BD1 domain and BD2 domain of human-derived BRD4 and BRDT with those of mouse-derived Brd4 and Brdt. Figure 1B shows that the identity of the BD1 domain protein sequence between human and murine BRD4 and BRDT is 78.082%, and there are 13 amino acid residues with similarity. The identity of the BD2 domain protein sequence is 69.863%, and there are 15 amino acid residues with similarity ( Figure 1B). These results show that the BD domains of BRD4 and BRDT from humans and mice are highly conserved.

Simulation analysis of the binding of BET family protein molecules to NHWD870 was then performed. The binding energy (BE) and binding affinity (BA) were calculated to be –145.625 KJ/mol and –9.30 kcal/mol, respectively ( Supplementary Figure S2A). Further simulation results showed that the interaction between BRDT and NHWD870 mainly occurs between aromatic rings and hydrogen and between aromatic rings and cations ( Figure 1C). We then calculated the IC _50_ of protein binding inhibition using a protein binding assay. Our results showed that the IC _50_ values of the binding of NHWD870 with BRD4 (BD1, BD2), BRD4 (BD1), and BRDT (BD1) were 0.64 nM, 0.34 nM, and 0.69 nM, respectively ( Figure 1D, and Supplementary Table S2), suggesting that NHWD870 could bind to BRDT as efficiently as to BRD4.

Further analysis of the mRNA expression data of Brd4 and Brdt in 20 types of spermatogenic cells from GEO [44] showed that Brd4 was expressed at low levels in early spermatogonia, as the FPKM value for Brd4 was <0.3 ( Figure 1E,F). Although the expression of Brdt was low in early spermatogonia, it showed its highest expression in the spermatocyte stage of meiosis ( Figure 1E,F). Further immunohistochemical staining results ( Supplementary Figure S2B) and the mRNA expression of Brd4 and Brdt in four different testicular cell lines (GC1, GC2, TM3, and TM4; Supplementary Figure S2C) verified these findings. Therefore, weak expression of Brd4 was only detected in spermatogonia. The expression of Brd4 in spermatocytes and cells at various stages after meiosis was almost undetectable. Furthermore, the expression of Brdt was detected in spermatocytes and sperm cells at all stages after meiosis. These results suggested that NHWD870 could potentially interact with Brdt in preference to Brd4 in the testis.

Taken together, the above results suggested that NHWD870 could interact with BRDT and potentially be used as a compound targeting the testis where BRDT is highly expressed.

### NHWD870 has contraceptive effects in mice

By validating four murine testicular cell lines (GC1, GC2, TM3, and TM4), NHWD870 produced inhibitory effects on the proliferation and apoptosis of different testicular cell lines *in vitro* ( Supplementary Figure S3). Therefore, we studied how NHWD870 delivers these contraceptive effects in mice.

**Figure 2:**
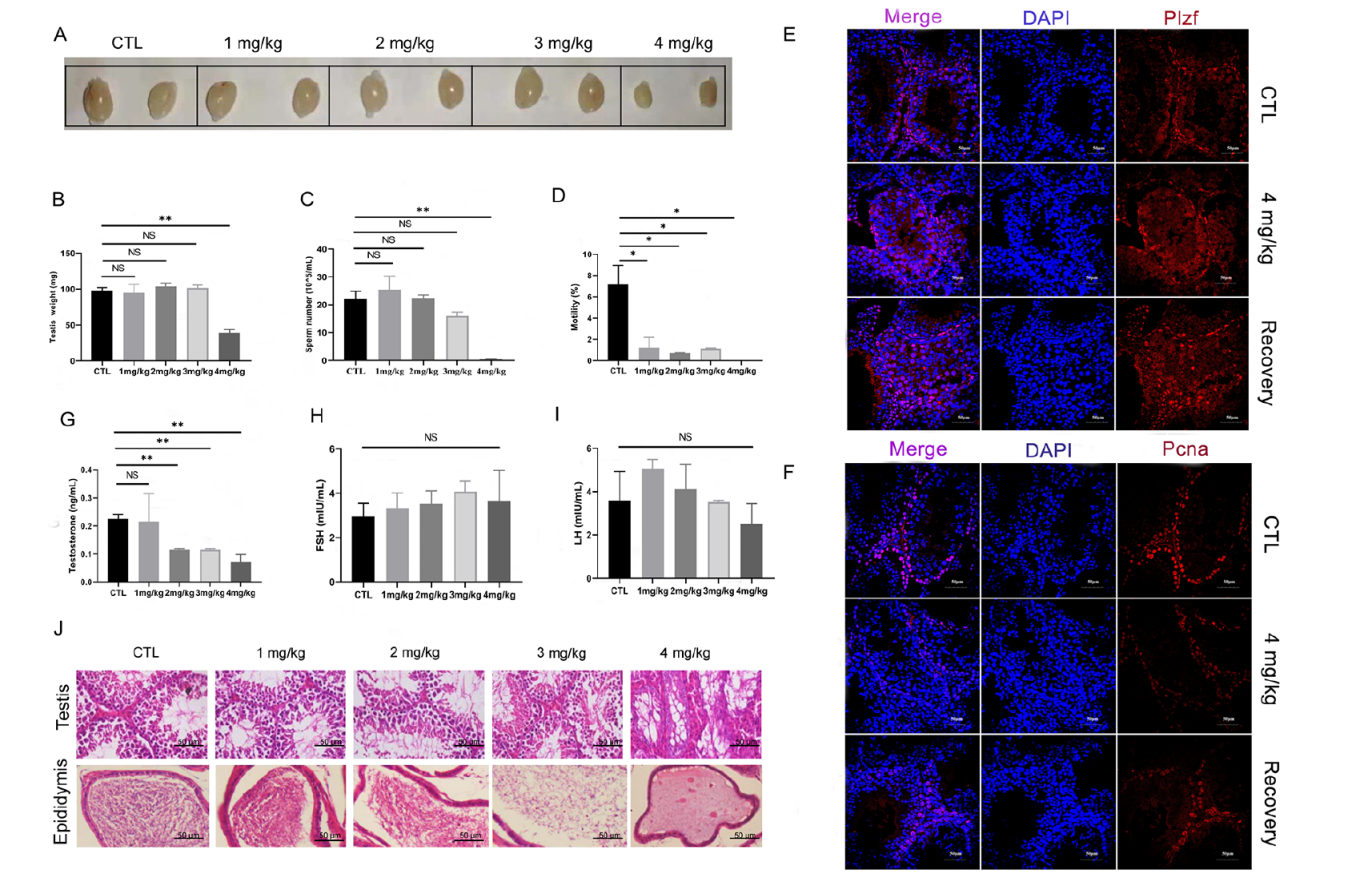
Figure 2 Effects of different doses of NHWD870 on the reproductive phenotypes of mice after 3 weeks of intragastric administration Different drug dosages were used (CTL, 1 mg/kg, 2 mg/kg, 3 mg/kg, and 4 mg/kg), and different parameters were examined. (A) Morphology of mouse testis. (B) Testis weight. (C) Sperm count. (D) Sperm motility ratio. (E,F) The effect of NHWD870 on mouse undifferentiated spermatogonia (Plzf) (E) and proliferating cell nuclear antigen (Pcna) (F) after administration of 4 mg/kg NHWD870 for 3 weeks and recovery of 4 mg/kg NHWD870 for 6 weeks. Magnification: 40×. Scale bar: 50 μm. Changes in hormone levels after NHWD870 administration. (G) Serum testosterone. (H) FSH. (I) LH. (J) Morphological changes in the testis and epididymis of mice after NHWD870 administration. Scale bar: 50 μm. The number of animals in each group, n=3; * P<0.05, ** P<0.01. NS, no significance.

After three weeks of NHWD870 administration, the testis and mature sperm of mice in each group were collected. The testicular morphology showed no abnormalities in each group compared with the control group ( Figure 2A). However, the testicular volume of the mice in the 4 mg/kg group was notably reduced by 50% ( Figure 2B). The results on the mature sperm number from the cauda epididymis showed that there was no difference among the 1 mg/kg, 2 mg/kg, and 3 mg/kg groups compared to the control, while there was a reduction of 70% in the 4 mg/kg group ( Figure 2C). The CASA results showed that sperm motility after treatment with 1 mg/kg, 2 mg/kg, and 3 mg/kg NHWD870 for 3 weeks was notably reduced to 1.22%, 0.70%, and 1.14%, respectively ( Figure 2D). When the administered dose reached 4 mg/kg, sperm motility was reduced to almost zero ( Figure 2D). Based on transcriptomic and proteomic results, the downregulated genes are normally involved in spermatogenesis and include genes related to the assembly of microtubules and cilia and ion channels on sperm membranes. The results indicate that even at the doses of 1/2/3 mg/kg, NHWD870 still significantly affected sperm motility because it may affect the expression of genes related to the assembly of microtubules and cilia, and genes related to ion channels on sperm membranes were also significantly affected.

To verify the effect of 4 mg/kg NHWD870 on mouse testicular tissue, the specific expression genes Ddx4 for spermatogonia ( Supplementary Figure S4A), Plzf for undifferentiated spermatogonia ( Figure 2E), Pcna ( Figure 2F) and Ki67 ( Supplementary Figure S5) for proliferating spermatogonia, and TUNEL for cell apoptosis in testicular tissue from mice ( Supplementary Figure S5) were used for immunofluorescence staining. The results showed that the remaining cells in the seminiferous tubules of mice were undifferentiated spermatogonia ( Figure 2E and Supplementary Figure S4A), indicating that NHWD870 produced no obvious damage to undifferentiated spermatogonia. By detecting Pcna and Ki67 for cell proliferation in testicular tissue from mice with NHWD870 treatment and after recovery ( Figure 2F and Supplementary Figure S5), we found that NHWD870 produced inhibitory effects on cell proliferation in testicular tissue, but it could recover after stopping dosing for 6 weeks. By detecting TUNEL for cell apoptosis in testicular tissue from mice with NHWD870 treatment and after recovery ( Supplementary Figure S5), we found that NHWD870 increased the cell apoptosis level in testicular tissue, but it also recovered after stopping dosing for 6 weeks, which is consistent with the results of cells *in vitro*. This meant that the mice still retained their fertility after medication and that the spermatogonia continued to differentiate and develop into mature sperm once drug administration was terminated.

We checked the changes in hormone levels after NHWD870 administration. Our results showed that there was no difference in the levels of FSH and LH among the groups ( Figure 2H,I). For the level of LH ( Figure 2I), because we only tested LH levels in three mice at each dose, the individual heterogeneity of mice was relatively large for the examination of hormone levels, which led to changes in LH levels that can be observed here, but there was no statistically significant difference. There was no difference in testosterone levels between the control group and the 1 mg/kg group. However, the testosterone levels among the other groups were all decreased, with a 50% drop for the 2 mg/kg and 3 mg/kg groups, and 32.57% for the 4 mg/kg group ( Figure 2G). This might be caused by NHWD870 targeting BRD4. We found that BRD4 is expressed in Leydig cells of the testis based on the Human Protein Atlas ( http://www.proteinatlas.org). According to immunofluorescence staining, the marker protein Cyp11a1 of Leydig cells was decreased after drug administration, but it recovered after stopping dosing ( Supplementary Figure S4B), indicating that Leydig cells were also damaged by NHWD870.

Staining of epididymal tissue sections revealed no difference in the number of sperm in the cauda epididymis among the control, 1 mg/kg, and 2 mg/kg groups. However, the number of sperm in the cauda epididymis was reduced in the 3 mg/kg group, and there were almost no sperm in the 4 mg/kg group ( Figure 2J). These results suggested that NHWD870 not only exerted its inhibitory effects by binding to Brdt spermatocytes and spermatids but also affected the process of sperm maturation.

### The contraceptive effect of NHWD870 in mice is reversible

**Figure 3:**
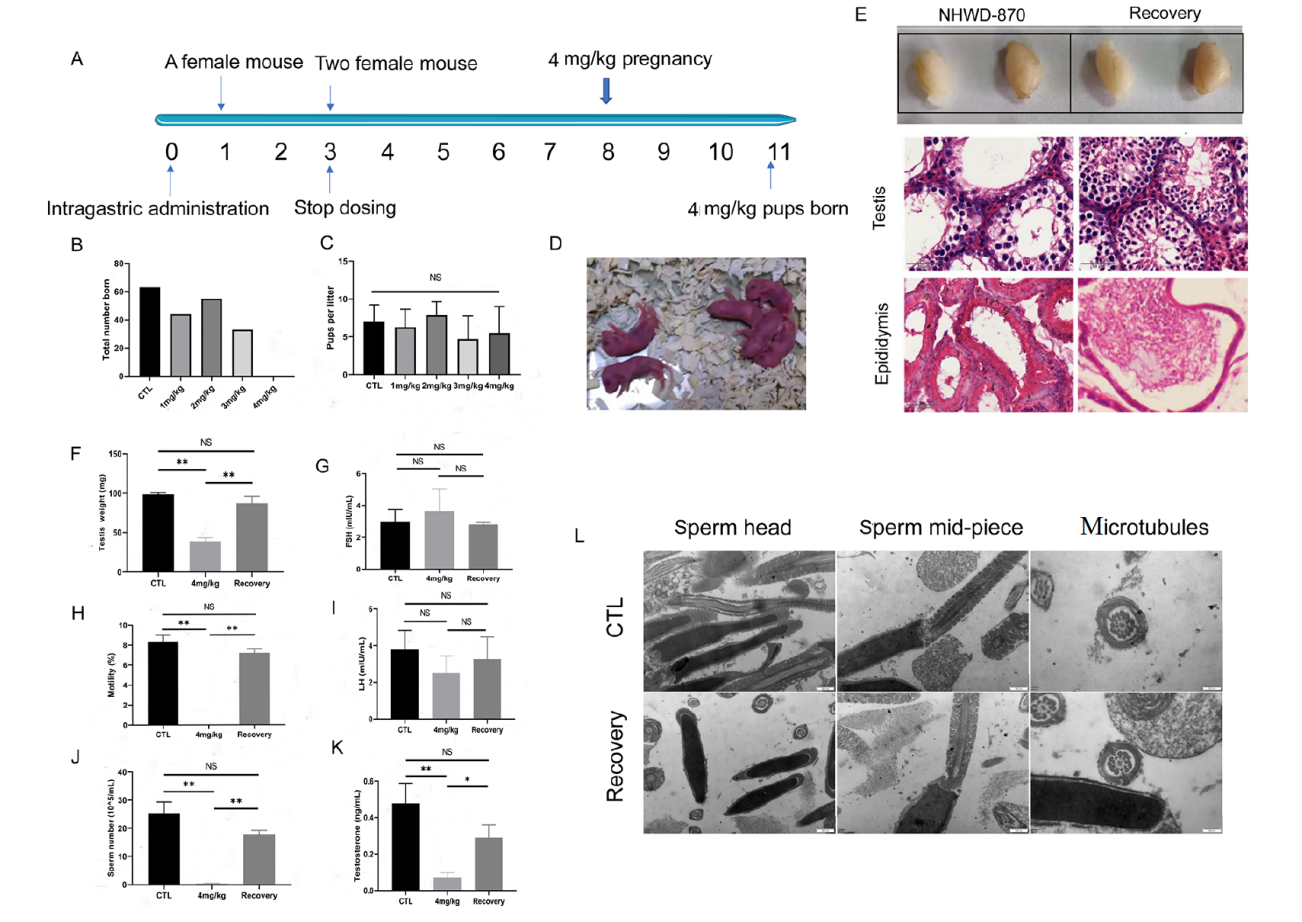
Figure 3 NHWD870 treatment led to a reversible contraceptive effect *in vivo* (A) Experimental protocol of different doses of NHWD870 administration and mating times. Female mice were introduced at the end of week 1 (one female mouse) and week 3 (two female mice) of the NHWD870 treatment. Female mice that were mated with 4 mg/kg NHWD870-treated male mice became pregnant at week 8, and healthy offspring were born at week 11. (B) The number of offspring in each group during the mating experiment. (C) The number of pups per litter produced in each group during the mating experiment. (D) Healthy offspring were born from 4 mg/kg NHWD870-treated mice, which indicated restored fertility. Comparison of the reproductive system of mice and changes in hormone levels between 3 weeks of 4 mg/kg NHWD870 treatment and after 8 weeks discontinuation of the treatment. (E) Testis, H&E section of the seminiferous tubules and epididymis from mice. Scale bar: 50 μm. (F) Testicular weight. (G) FSH level. (H) Sperm motility ratio. (I) LH level. (J) Sperm count. (K) Testosterone level. (L) Transmission electron microscopy (TEM) shows that there were no obvious changes in the sperm ultrastructure between the recovery group and the control group. Magnification: 30000×. Scale bar: 500 nm. The number of animals in each group, n=3, * P<0.05, ** P<0.01. NS, no significance.

We designed a mating experiment to test whether the contraceptive effect of NHWD870 in mice is reversible. In this experiment, the mice were treated with different doses of NHWD870 for 3 weeks as mentioned above. Female mice were introduced on week 1 (one female mouse) and week 3 (two female mice) of the NHWD870 treatment period. The total number of fertile matings of each group of female mice was counted for 8 weeks after the termination of NHWD870 treatment at the end of week 3 ( Figure 3A).

During this period, the number of offspring produced from the 1 mg/kg and 2 mg/kg treated groups decreased slightly. The offspring born from the 3 mg/kg group decreased by 50%, and no offspring was born from the 4 mg/kg group, indicating that the 4 mg/kg dose of NHWD870 had a good contraceptive effect ( Figure 3B). However, at 8 weeks after termination of the NHWD870 treatment, healthy offspring were born from the 4 mg/kg group. The pregnancy cycle of mice was approximately 3 weeks; therefore, mice from the 4 mg/kg group resumed their fertility as early as 6 weeks after termination of NHWD870 treatment. In addition, there was no difference in the number of pups per litter among the groups treated with NHWD870 ( Figure 3C,D).

We retrieved testes and sperm from mice treated with 4 mg/kg NHWD870 for the first 3 weeks and then after a recovery of week 8. Compared with the control group, the weight of the testis of the mice returned to normal ( Figure 3F). The sperm count also recovered to 60% of the normal level ( Figure 3J), and the sperm motility ratio returned to close to that of normal mice ( Figure 3H). We further compared the testis and cauda epididymis tissue sections from these samples. After 6 weeks of recovery, spermatogenesis in the seminiferous tubules of the mice was restored, and spermatids and sperm cells of subsequent stages were found ( Figure 3E). There was also an accumulation of mature sperm in the cauda epididymites, indicating that spermatogenesis and fertility had recovered well ( Figure 3E). The serum levels of sex hormones were also determined, and the results showed that the FSH and LH levels had returned to normal ( Figure 3G,I). The testosterone levels were still lower than those of the control group, but they were already higher than those at the time when NHWD870 was first administered ( Figure 3K). We compared the sperm ultrastructure between the control and recovery groups with TEM. There were no obvious changes in sperm head, sperm mid-piece or microtubes between the control and recovery groups ( Figure 3L and Supplementary Figure S6), suggesting that sperm recovery works well after drug withdrawal.

In the process of intragastric administration in our study, compared with that of the normal mice, the body weight of mice treated with the 3 mg/kg and 4 mg/kg NHWD870 dropped notably within 7 days of treatment. The body weight and H&E staining results showed that intragastric administration of NHWD870 caused a certain degree of liver damage and weight loss in mice ( Supplementary Figure S7A,B); however, subsequently, the body weight gradually returned to normal ( Supplementary Figure S7A,B). However, no side effects on other tissues and organs were observed in our study. To reduce the side effects of hormonal male contraceptives and facilitate their use, it is normally considered important to test different administration routes for the drug. Considering the low drug tolerance of mice and to verify the efficacy of NHWD870 in different species, we used SD rats, which have better drug tolerance, as our additional experimental animals to test how different administration routes would affect the contraceptive effect of NHWD870. We performed three different administrations (4 mg/kg NHWD870 by gavage, 1 mg/kg NHWD870 by tail vein injection, and 4 mg/kg NHWD870 by subcutaneous injection) and investigated their effects on the liver, testis, and epididymis. H&E sections showed no difference in the effect of NHWD870 on the rat liver between the subcutaneous administration methods and the control group ( Supplementary Figure S7C). By examining sperm in the H&E-stained sections of rat epididymis, we counted an average of 237 sperm in each chamber of the epididymis of the control group, 148 for the tail vein injection group, and 138 for the gavage group. The number of mature sperm in the cauda epididymis of rats treated with NHWD870 through tail vein injection was decreased to 62%; for gavage, it was decreased to 58%; and no mature sperm were observed for the subcutaneous injection group ( Supplementary Figure S7C), indicating that subcutaneous injection of NHWD870 may be a better way to achieve contraception.

Taken together, the above results indicated that the contraceptive effect of NHWD870 in mice is reversible and that NHWD870 does not produce any long-term effects on testis physiology or reproductive capacity.

### Molecular analysis revealed the potential pathway for contraceptive effects in NHWD870-treated testes

The contraceptive effects of NHWD870 were assumed to be largely due to its role as a novel BRDT inhibitor, similar to that of JQ1 [31]. However, the detailed mechanism still requires further investigation. The study using NHWD870 in treating cancer has shed some light on the general mechanism of how inhibition of BRD4 affects the regulation and interaction of gene expression [36]. Here, we carried out transcriptomics ( Supplementary Figure S8A,B) and proteomics ( Supplementary Figure S9) analyses in 4 mg/kg NHWD870-treated testes to explore the role of NHWD870 in the process of inhibiting spermatogenesis and sperm maturation.

**Figure 4:**
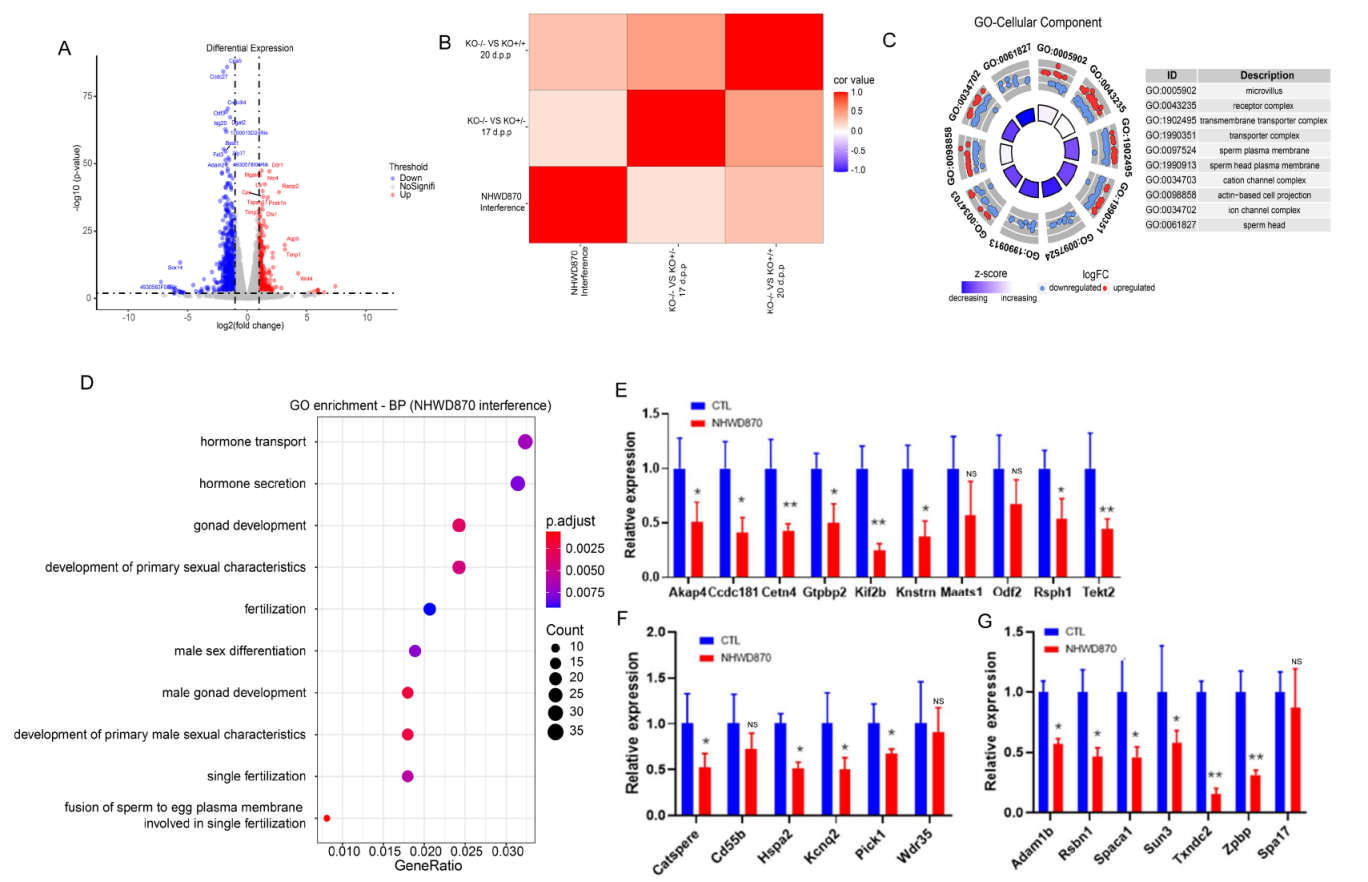
Figure 4 Transcriptomics analysis of NHWD870-treated murine testes showed the enriched pathways affected by drug treatment (A) Volcano map analysis of differentially expressed genes, which displayed 396 upregulated and 820 downregulated genes. Padj≤0.05, |log2FC|≥1. (B) Correlation of gene expression profile changes among KO ‒/‒ vs KO +/+, KO ‒/‒ vs KO +/‒, and NHWD870 drug intervention groups. Padj≤0.05, |log2FC|≥1. (C) Chord plot of the top 10 enriched GO terms of cellular component (cell component). Padj≤0.01, |log2FC|≥1. (D) GO terms related to fertility in the NHWD870 group (biological process). Padj≤0.01, |log2FC|≥1. (E) Expression of genes related to microtubule assembly and ciliary movement. (F) Sperm ion channel-related gene expression. (G) Spermatogenesis-related gene expression. Actin was used as an internal reference gene for qPCR. The number of animals in each group, n=3, * P<0.05, ** P<0.01. NS, no significance.

Our data revealed 1216 differentially expressed genes, including 396 upregulated and 820 downregulated genes in the testis between the control group and NHWD870 group ( Figure 4A). We further investigated whether the dysregulated gene expression is correlated with BRDT function during spermatogenesis. Combined with the existing transcriptomics data of GSE39909 of BRDT knockout mice ( Supplementary Table S3), we performed a correlation analysis of the expression profiles of GSE39909 and our RNA-seq samples ( Supplementary Figure S8C,D). Within the two data sets, the correlation of the expression profiles of samples between groups was high (>0.8), indicating that BRDT in the testis, as a major NHWD870 target, has a relatively specific effect on spermatogenesis-related pathways based on the expression profiling of the entire transcriptome. Therefore, we focused on those genes that are significantly differentially expressed in the two data sets.

Using the transcriptomics data of GSE39909 from BRDT knockout mice, we further calculated the correlation of gene expression profile changes between KO–/– vs KO+/+ (group 1), KO–/– vs KO+/– (group 2), and NHWD870 drug intervention (group 3); log2FC data were positively correlated in all three groups ( Figure 4B), indicating that BRDT knockout and NHWD870 drug intervention had similar effects on overall gene expression changes.

Differentially expressed genes were screened based on the cut-off criteria of *P*<0.01 and |log2FC| ≥1. The results of the comparison among the three groups are graphically displayed by a Venn diagram in Supplementary Figure S7A, which assesses the overlap and difference of differentially expressed genes in the three groups. Group 3 (NHWD870 treatment group) contained the most differentially expressed genes. In the three data sets, the number of differentially expressed genes that were downregulated was far greater than that of upregulated genes, and the differentially expressed genes between group 1 and group 2 were more similar ( Supplementary Figure S10A).

Using *P*adj ≤0.01 as the threshold, GO analysis was conducted, and the results showed that cellular components were enriched in 41 pathways that were involved in microvilli, sperm plasma membrane, and sperm head ( Figure 4C). Interestingly, GO-molecular function ( Supplementary Figure S10B) analysis showed that most molecular changes were enriched in ion channels, indicating that these genes are possibly the transcriptional targets of Brdt in the testis. Pathway analysis showed that the differentially expressed genes of the NHWD870 drug intervention group were enriched in 68 pathways, of which the pathways related to fertility are shown in Figure 4D. These genes are normally involved in hormone transport, spermatogenesis, the assembly of microtubules and cilia, and ion channels on sperm membranes.

By using real-time PCR, we verified the differential genes associated with spermatogenesis-related pathways, and they were found to be genes related to sperm production, including *Spaca1*, *Sun3*, and *Txndc2*; *Akap4*, *Cetn4*, *Odf2*, and *Tekt2* are related to cilia assembly and movement; and ion channels are related to the gene CatSper. The abovementioned spermatogenesis and sperm maturation genes, including sperm production, ciliary motility ( Figure 4E,G), and ion channels ( Figure 4F), were downregulated in NHWD870-treated mice.

**Figure 5:**
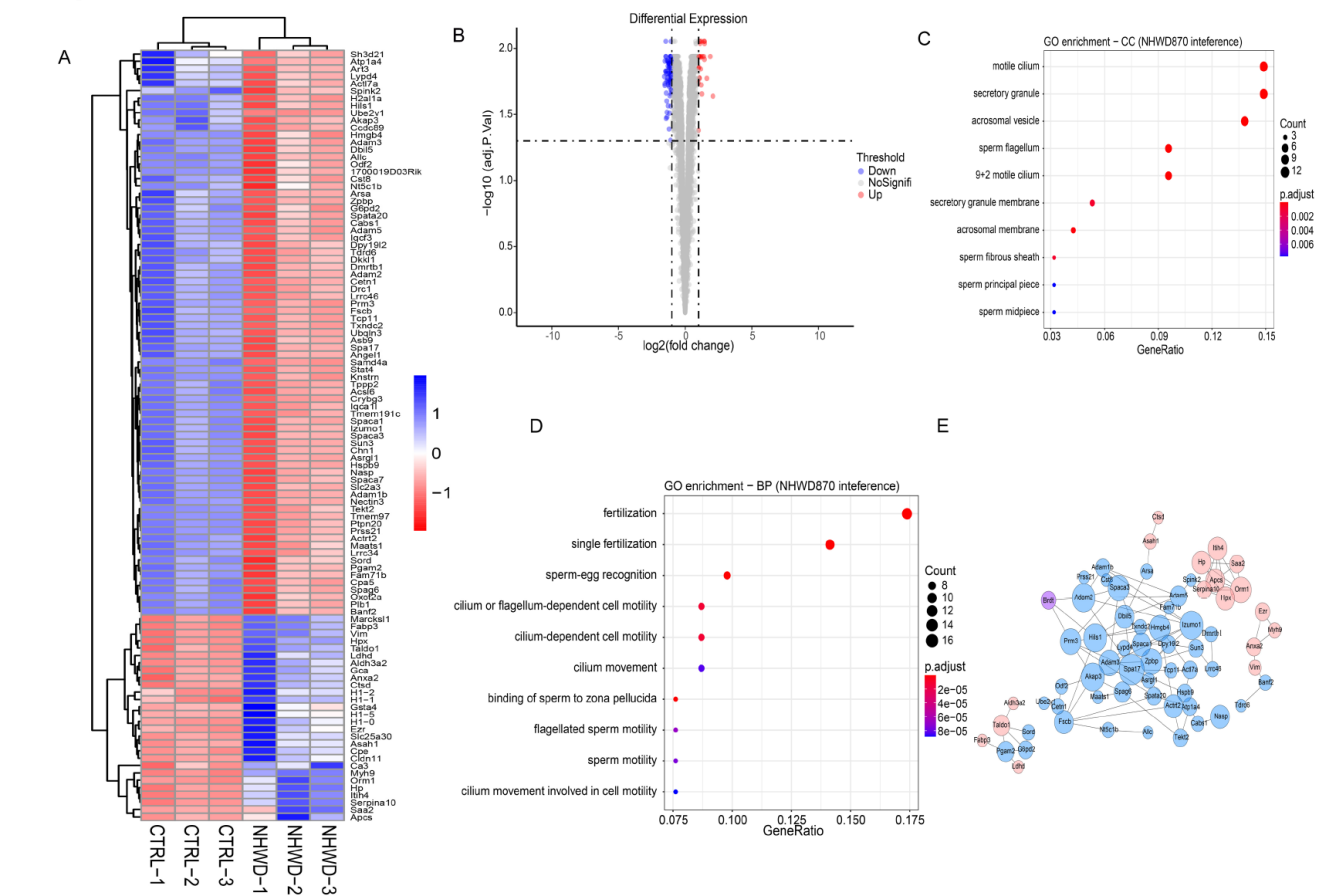
Figure 5 Proteomics analysis of NHWD870-treated murine testes revealed the enriched pathways affected by drug treatment (A) Heatmap of differentially expressed proteins in NHWD870-treated mouse testes. Red and blue colors indicate whether the expression value is above (blue) or below (red) the mean expression value across samples (the data were normalized from −1 to +1). n=3 for each group. Padj≤0.05, |log2FC|≥1. (B) Volcano map analysis of differentially expressed proteins, with 28 upregulated and 77 downregulated proteins. Padj≤0.05, |log2FC|≥1. (C) The top ten GO items (cell component) with the highest significance. Padj≤0.01, |log2FC|≥1. (D) The top ten GO items (biological process) with the highest significance. Padj≤0.01, |log2FC|≥1. (E) Protein‒protein interaction network diagram. Red indicates upregulation; blue indicates downregulation.

Proteomic analysis was conducted in the testes of NHWD870-treated mice and control mice. Based on the cut-off criteria of *P*adj≤0.05 and |log2FC|≥1, a total of 105 differentially expressed proteins were obtained, of which 28 were upregulated and 77 were downregulated ( Figure 5A,B), indicating that NHWD870 led to the downregulation of more proteins. A total of 21 genes showed significant differential expression at the transcription level and protein level, of which 20 genes had the same up- and downregulation trend at both the transcription level and protein level ( Supplementary Figure S11). They were Act17a, Akap3, Allc, Asb9, Banf2, Chn1, Cldn11, Cpa5, Cpe, G6pd2, Hp, Lzumo1, Ldhd, Plb1, Serpina10, Spaca3, Spag6, Spata20, Stat4, and Ubqin3. According to GO analysis, abnormal expression of these genes is reported to seriously affect spermatogenesis and maturation, leading to infertile phenotypes in mice.

Using *P*adj≤0.01 as the threshold, the differentially expressed proteins were enriched in a total of 40 pathways. The top ten most significant pathways in the cell component and biological process categories are shown in Figure 5C,D, which mainly involve sperm-related, 9+2 motile cilium (cell component) and fertilization-related pathways (biological process). A protein–protein interaction network for differentially expressed proteins was drawn using STRING v11.5 [45], and the results are shown in Figure 5E. Western blot analysis further verified the selected downregulation of markers for related pathways, including CEP112 for spermatogenesis, Spata6, Wdr60, and AKAP3 for microtubule assembly, ciliary movement, and Na/K-ATPase in ion channels ( Supplementary Figure S12). Therefore, the gene expression of spermatogenesis, microtubule assembly, ciliary movement, and ion channels in the testis of mice treated with NHWD870 was downregulated, which could be responsible for the reduced fertility of the mice.

## Discussion

In this study, we demonstrated that the novel BRDT inhibitor NHWD870 is an excellent candidate molecule as a male contraceptive. The molecular interaction of NHWD870 and BRDT was investigated through simulation and protein binding assays. The inhibitory effects of NHWD870 on spermatogenesis in male mice were investigated by *in vivo* animal experiments. Our results strongly suggest that NHWD870 can be used as a small molecule lead compound that specifically targets the BRDT protein in the testis to produce contraceptive effects by affecting spermatogenesis and androgen secretion.

High binding energy and binding affinity are found in the binding of NHWD870 with BRD4 and BRDT. Although NHWD870 was invented as a BRD4 inhibitor, due to the high conservation of BRDT and BRD4 sequences, NHWD870 can also inhibit BRDT both *in vitro* and *in vivo*. As BRDT is highly expressed in the testis, we therefore focused on BRD4 and BRDT. Nevertheless, it cannot be ruled out that NHWD870 may also work through inhibition of other members of the BET subfamily that are also expressed in murine testes [31].

As an analog of JQ1, NHWD870 shows several advantages over JQ1 [36]. First, *in vitro* protein binding assays showed specific binding of NHWD870 with the BD1 and BD2 domains. Second, NHWD870 effectively inhibits germ cells. *In vivo*, NHWD870 requires a low dosage to be effective, whereas 50 mg/kg JQ1 needs to be injected intraperitoneally for 6 weeks to produce contraception in mice. This usage of JQ1 reduced testicular volume to 50% after 6 weeks of continuous administration [31], while only 4 mg/kg NHWD870 guaranteed a reversible contraceptive effect after only 3 weeks of administration in our study. Third, a shorter discontinuation recovery period (6 weeks) is needed for NHWD870 compared with a 4-month recovery period to produce healthy offspring for JQ1 [31]. Fourth, a more convenient method of oral administration makes NHWD870 more amenable for translation. NHWD870 is more effective than JQ1, and the oral route of administration produces better subject compliance. Furthermore, our transcriptomics and proteomics analyses revealed that that NHWD 870 targets several key steps of sperm maturation.

Previous studies reported that non-hormonal contraceptives have side effects of weight loss in mice [46] and can cause damage to the liver and further affect the weight of animals, as the liver is the main site of drug metabolism [47]. Previous animal studies showed that many BET inhibitors cause various degrees of body weight loss, which are commonly used as a surrogate for gastrointestinal toxicity for drug treatment in animal models [ 48, 49] . To this end, we examined the effects of NHWD-870 on body weight changes and found that NHWD870 caused approximately 6%–8% body weight loss, which is considered acceptable and much less severe than BMS 986158, which led to approximately 11% body weight loss [36].

Although NHWD870 showed slight side effects of weight loss and liver toxicity in mice owing to its oral administration, no side effects on other tissues or organs were observed in our study. Compared with the gavage group, subcutaneous injection is more conducive to the absorption of fat-soluble drugs, and we found that the subcutaneous administration group had fewer side effects and better contraceptive effects than the gavage group. The results in the gavage group also indicated that future applications, such as microneedle drug delivery or a hormone-like subcutaneous implant, may represent better choices [ 50, 51] . In addition, the molecular docking results in this study highlighted the directions for improving the structure of small molecules based on drawn electrostatic maps near the interaction site ( Supplementary Figure S13). Subsequent structural optimization may be facilitated based on this observation.

In summary, in this study, we showed that the novel BET protein family inhibitor NHWD870 is highly effective in inhibiting and allowing the recovery of fertility in mice. It therefore has great potential as a contraceptive targeting BRDT.

## Data Availability

The transcriptomics data have been submitted to the SRA database. The number is PRJNA765522. The proteomics results have been submitted to Proteome X change via the PRIDE database. The number is PXD028773.

## Supporting information

139Supplyment_upload

## Supplementary Data

Supplementary data is available at *Acta Biochimica et Biophysica Sinica* online.
