# Supplementary material for: The novel BRDT inhibitor NHWD870 shows potential as a male contraceptive in mice: A novel inhibition of BRDT for male contraception
Source: Acta Biochim Biophys Sin (Shanghai). 2022 Sep 30;54(12):1789–800. doi: 10.3724/abbs.2022135 (PMC10157631; doi:10.3724/abbs.2022135)
Supplement: 139Supplyment_upload [file 139Supplyment_upload.pdf]

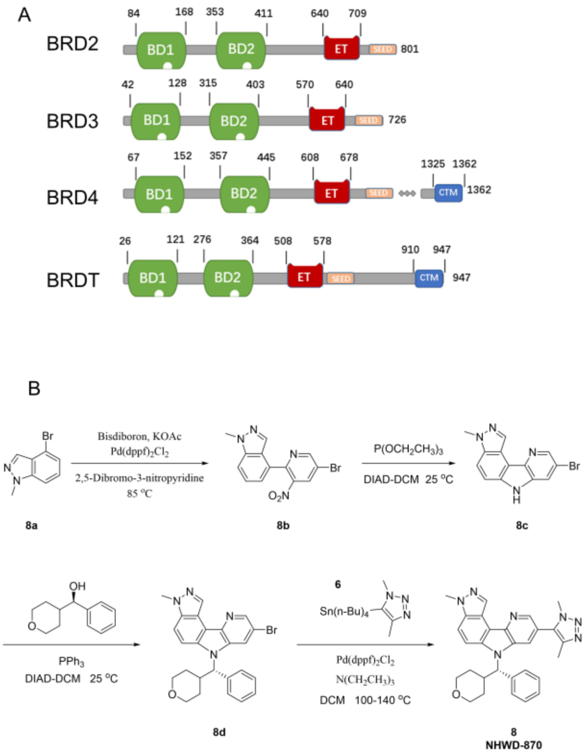

Supplementary Figure S1. **The diagram of BET family protein domain and NHWD870 synthesis** (A) The BET family protein structure. (B) The molecular synthesis route of NHWD870.

A

	Binding Energy (KJ/mol)		Binding Affinity (kcal/mol)	
	BD1	BD2	BD1	BD2
BRDT	-145.625	-128.420	-9.3	-8.56
BRD4	-137.584	-124.581	-7.59	-9.30
BRD3	-92.551	-130.227	-7.38	-9.06
BRD2	-126.394	-109.095	-9.28	-8.76

Force : Amber ff14SB/GAFF
TITP3P water model

B

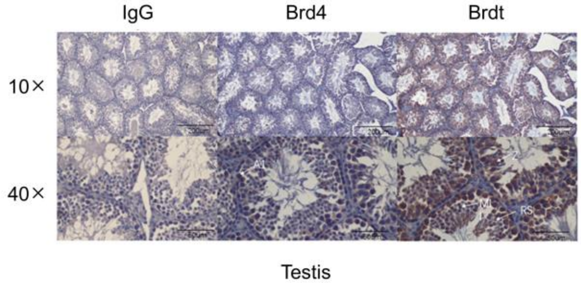

C

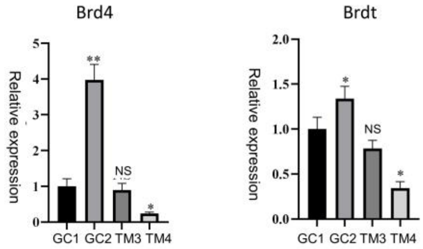

Supplementary Figure S2. Prediction of mouse BET family protein and NHWD870 binding and the expression of mouse BET family protein in testis (A) The binding energy and binding affinity of mouse BET family protein and NHWD870 were calculated after 50ns molecular dynamics simulation. Among them, the binding energy and binding affinity of NHWD870 on BRDT BD1 and BD2 domains were the lowest. This showed that the binding between NHWD870 and BRDT was the tightest. (B) Expression of Brd4 and Brdt in testis. A1--A1 type spermatogonia, Z--meiotic even-line spermatocytes, MII--meiotic II metaphase spermatids, RS--round spermatids. Magnification: 10×, scale bar: 200 μm, Magnification: 40×, scale bar=50 μm. (C) The expressions of Brd4 and Brdt mRNA in GC1, GC2, TM3 and TM4 cells, compared with the expression levels in GC1 cells. *Actin* was used as an internal reference gene.

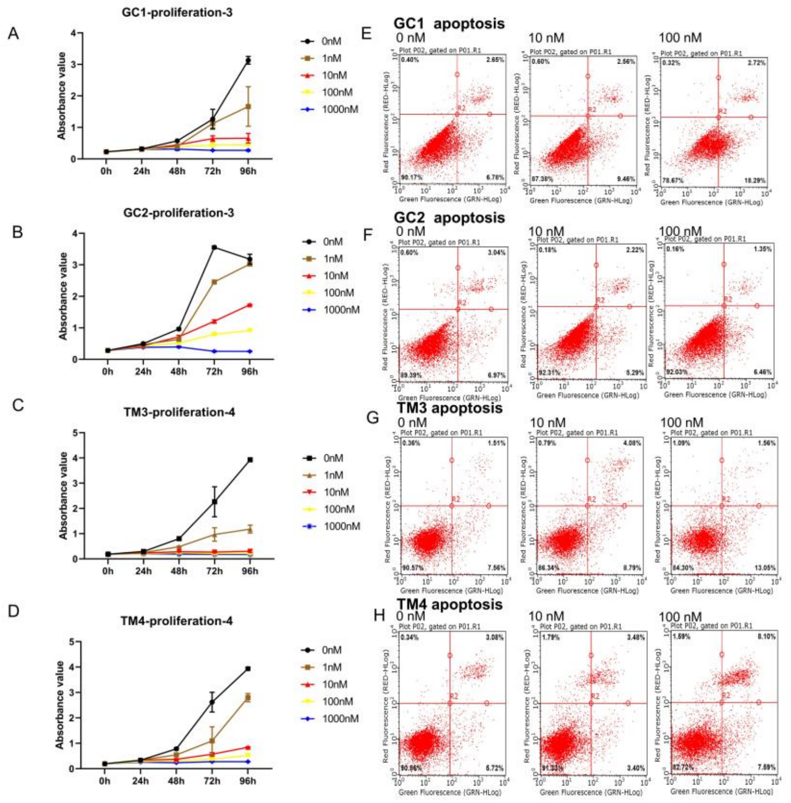

Supplementary Figure S3. **NHWD870 inhibits the cell proliferation and promotes apoptosis in different testicular cell lines** NHWD870 was found to have a significant inhibitory effect on GC1 (A), TM3 (C) and TM4 (D) cells at a concentration of 10 nM, and a significant inhibitory effect on GC2 cells (B) at a concentration of 100 nM. The effect of NHWD870 on apoptosis of different testicular cell lines of GC1 (E), GC2 (F), TM3 (G) and TM4 (H). NHWD870 showed pro-apoptotic effects on GC1, GC2, TM3 and TM4 cells in a dose-dependent manner. The percentage of early apoptotic cells in GC1 and TM3 were increased from 6.78% to 18.29%, and from 7.56% to 13.04% respectively when treated with 100 nM NHWD870.

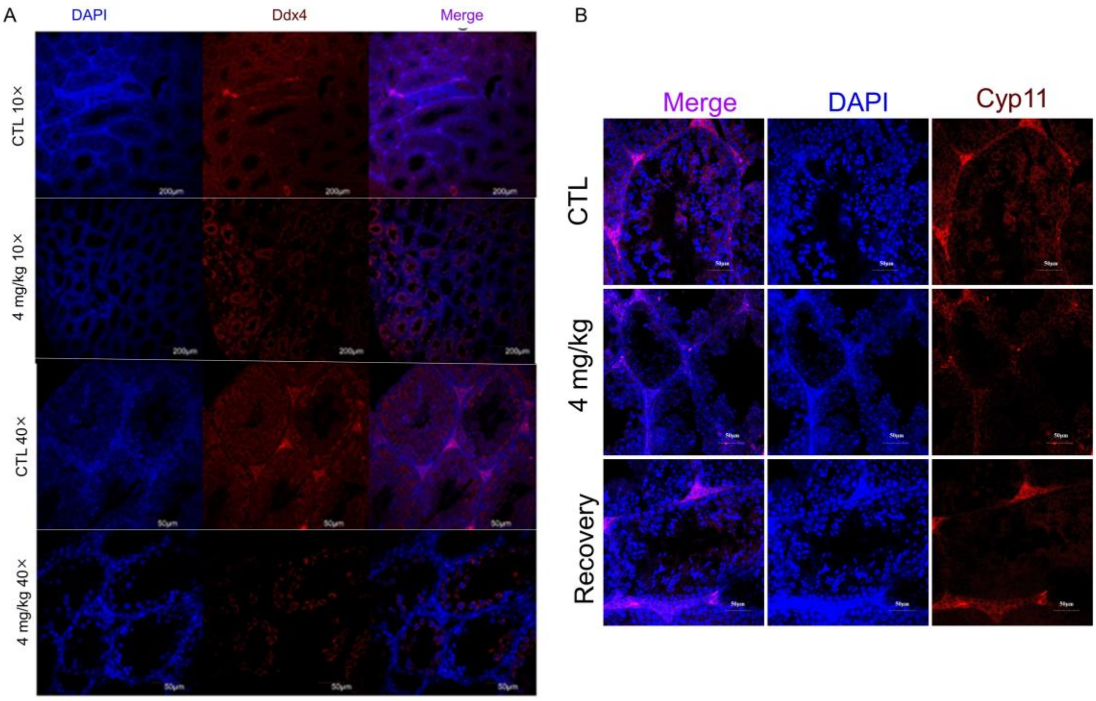

Supplementary Figure S4. Effect of NHWD870 on mouse spermatogonia and Leydig cells (A) The effect of NHWD870 on mouse spermatogonia (Ddx4) after administration with 4 mg/kg for 3 weeks. Magnification: 10×, scale bar: 200 μm. Magnification: 40×, scale bar: 50 μm. (B) The effect of NHWD870 on mouse Leydig cells (Cyp11a1) after administration with 4 mg/kg NHWD870 for 3 weeks and recovery of 4 mg/kg NHWD870 for 6 weeks. Magnification: 40×, scale bar: 50 μm.

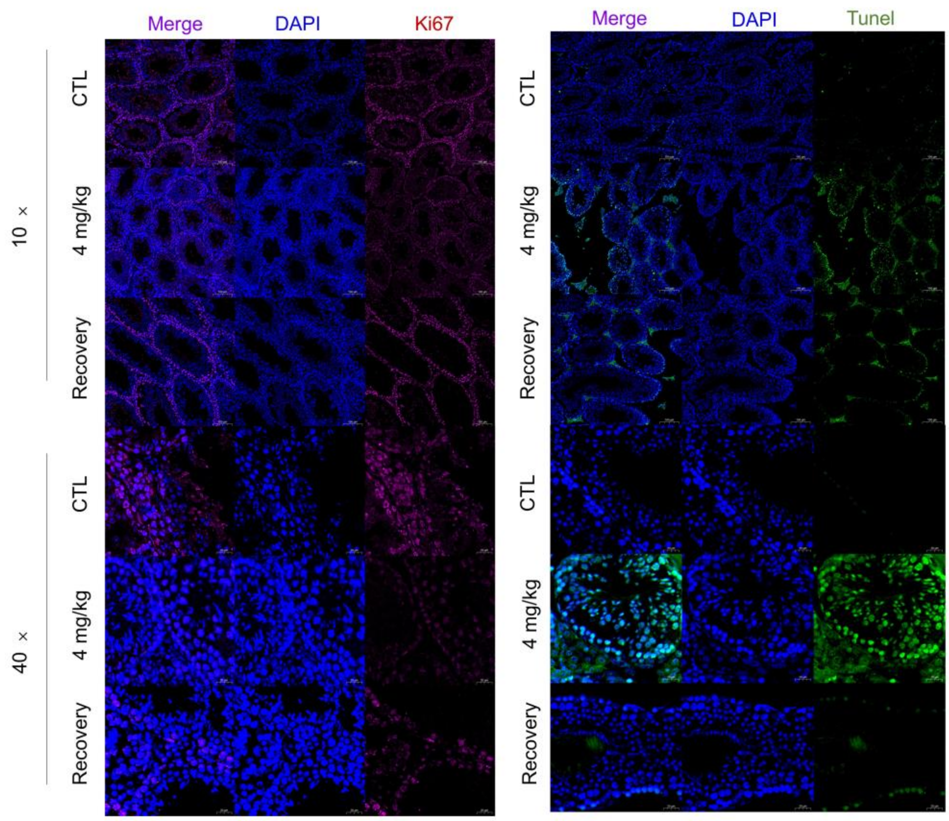

Supplementary Figure S5. NHWD870 inhibits the cell proliferation and promotes apoptosis in testicular tissue from mice The effect of NHWD870 on the cell proliferation (Ki67 staining) and the cell apoptosis (Tunel) in testicular tissue from mice after administration with 4 mg/kg NHWD870 for 3 weeks and recovery of 4 mg/kg NHWD870 for 6 weeks. Magnification: 10×, scale bar: 200 μm. Magnification: 40×, scale bar: 50 μm.

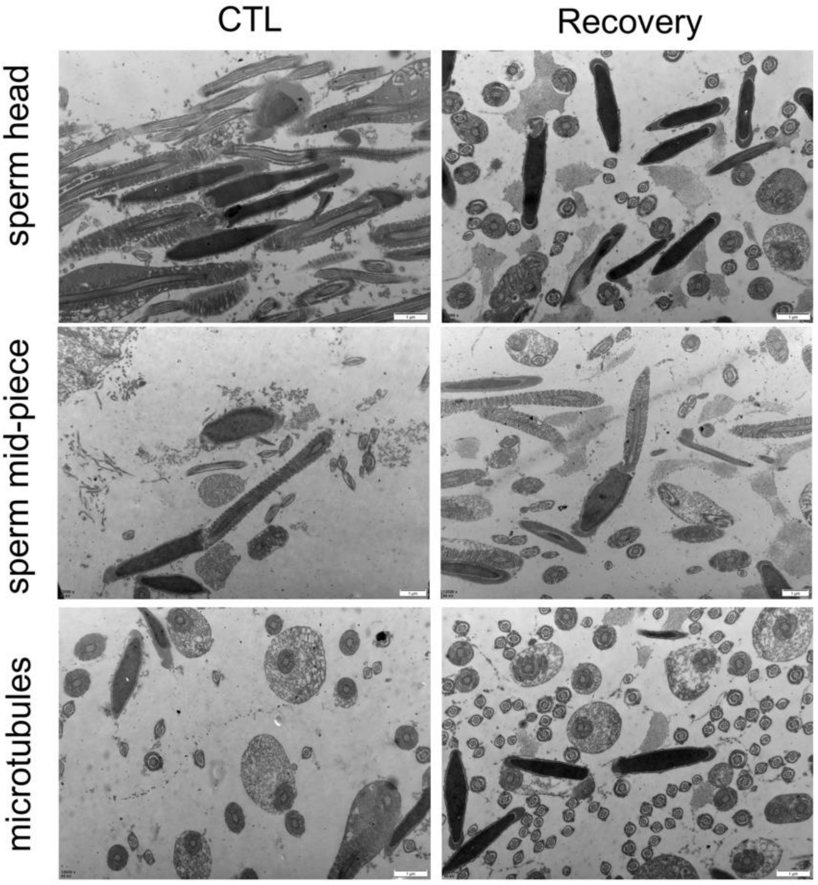

Supplementary Figure S6. The ultrastructure of the sperm between recovery group and control group Transmission electron microscopy (TEM) shows there were no obvious changes in the sperm ultrastructure (sperm head, sperm mid-piece and microtubes) between recovery group and control group. Magnification: 15000×, scale bar: 1 μm.

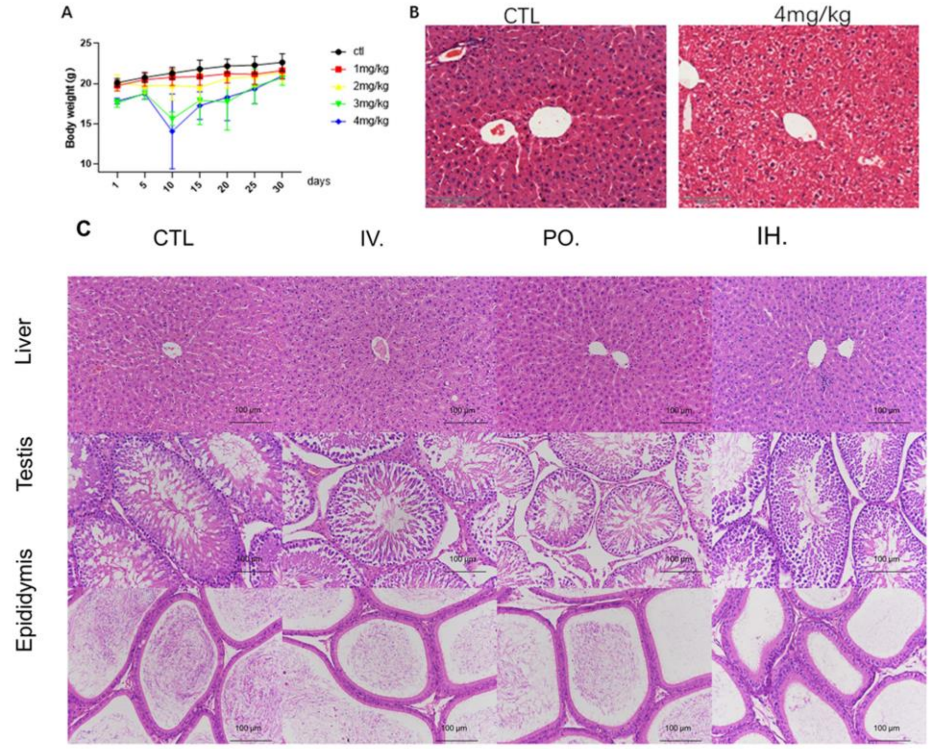

Supplementary Figure S7. The side effect of NHWD870 in mice and rats (A) The effect of different doses of NHWD870 on the body weight of mice. (B) The effect of NHWD870 on the liver of mice. (C) Effects of different administration methods of NHWD870 on liver toxicity and reproductive system in rats. Scale bar: 100 μm. The number of animals in each group, n=3.

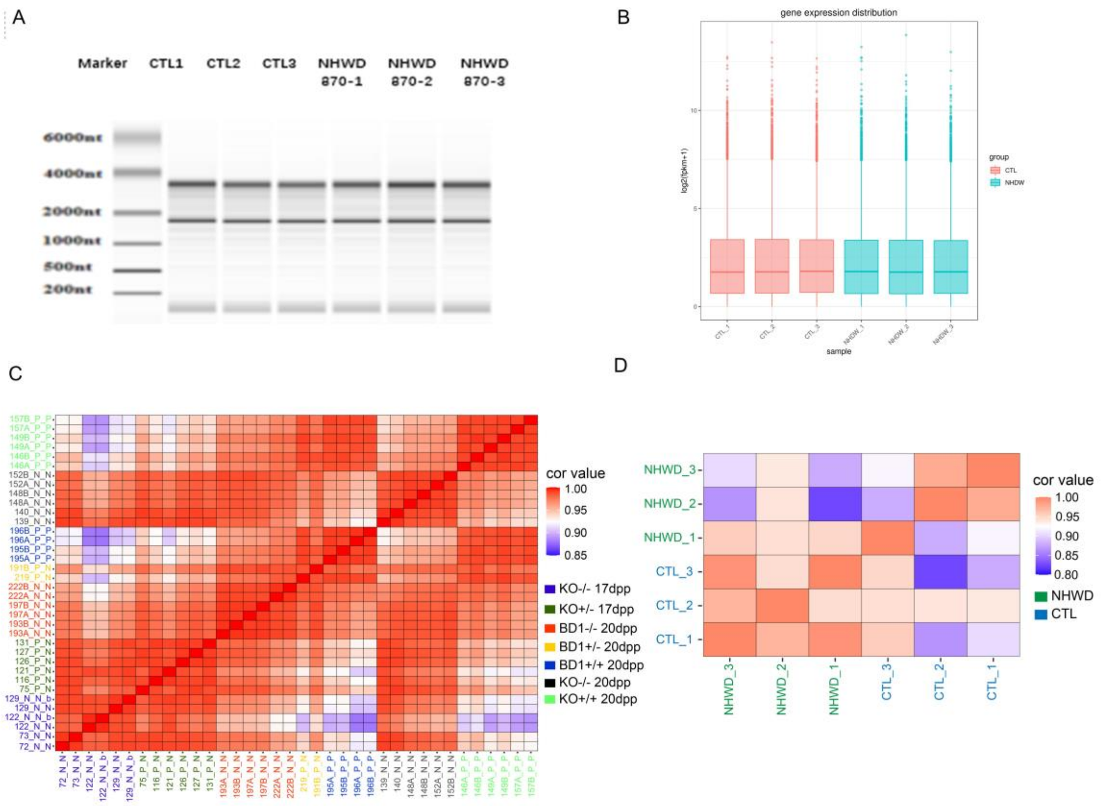

Supplementary Figure S8. Quality control, reference genome alignment and quantitative analysis of testicular transcriptome sequencing data (A) Agarose gel electrophoresis of RNA in testis tissue of mice in the control group and the NHWD870 group. (B) The distribution of gene expression in the testis tissue of mice in the control group and the NHWD870 group. (C) The correlation of expression profiles in the GSE39909 dataset. (D) The correlation of expression profiles in the NHWD870 interference RNA-seq dataset.

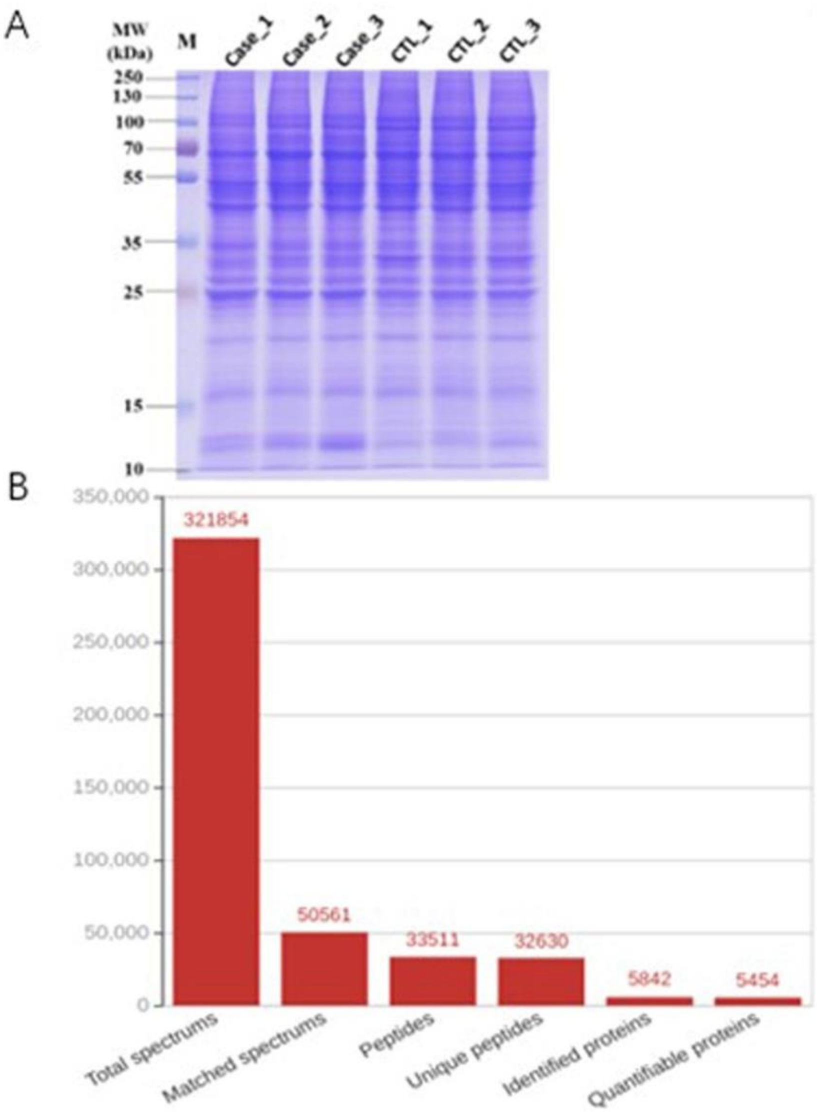

Supplementary Figure S9. Protein sample quality and mass spectrometry data quality control (A) SDS-PAGE gel image. After total protein was extracted from the testis tissues of normal mice and mice treated with 4 mg/kg NHWD870 for three weeks, proteomics analysis was conducted to study the effect of NHWD870 on spermatogenesis in mouse testes. A total of 15 μg of each sample was subject to SDS-PAGE and Coomassie brilliant blue staining. (B) Basic statistics of mass spectrum data. A total of 5842 proteins were identified in the testis of the NHWD870 group and the control group, of which 5454 could be quantified.

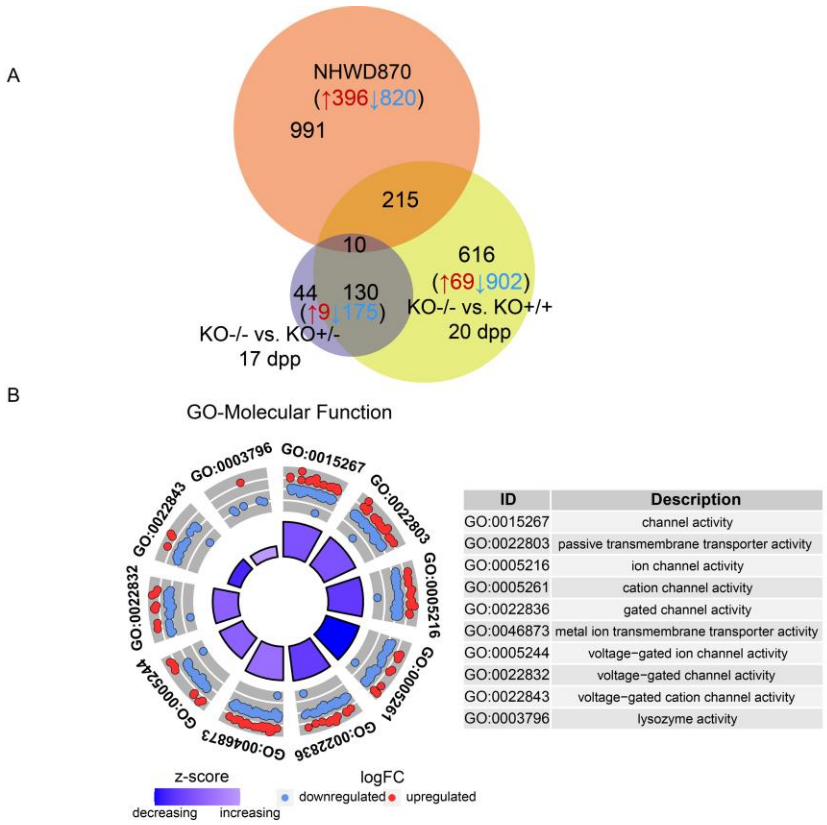

Supplementary Figure S10. GO enrichment of the differently expressed genes in the NHWD870 interference RNA-seq dataset (A) Venn diagram of three data sets among KO-/- vs KO+/+, KO-/- vs KO+/- and NHWD870 drug intervention groups. (B) Chord plot of top 10 enriched GO terms of molecular function (MF).

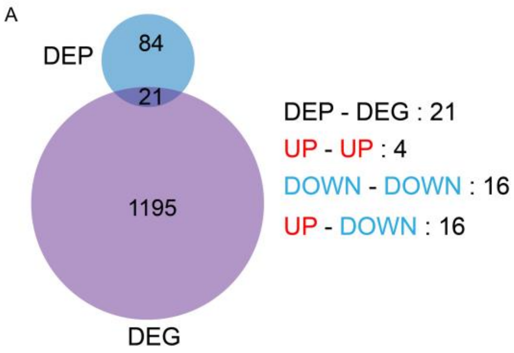

Common Genes	
1700019D03Rik	Hp
Actl7a	Izumo1
Akap3	Ldhd
Allc	Plb1
Asb9	Sperpina10
Banf2	Spaca3
Chn1	Spag6
Cldn11	Spata20
Cpa5	Stat4
Cpe	Ubqin3
G6pd	

Supplementary Figure S11. The situation of differentially expressed proteins and differentially expressed genes A total of 21 genes are differentially expressed at both the transcription and protein levels.

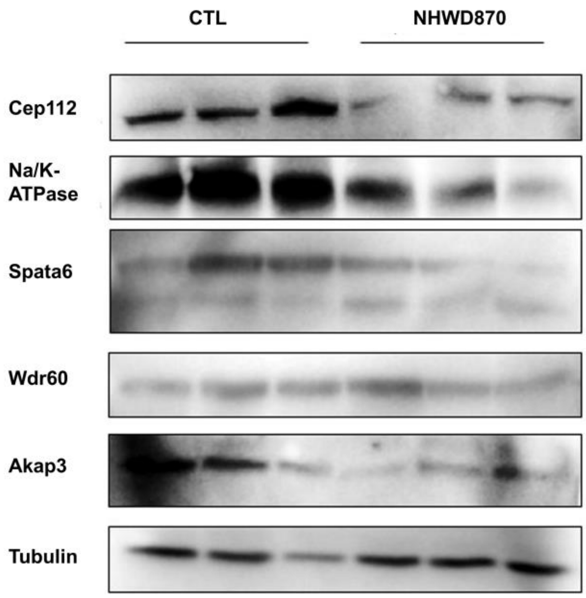

Supplementary Figure S12. Protein expressions of CEP112, Spata6, Wdr60 and AKAP3 for spermatogenesis, microtubule assembly and ciliary movement, and Na/K-ATPase for ion channels verified by western blot analysis The results of are consistent with the change trend of the protein group. Tubulin was used as the loading reference.

A
BRDT-BD1

B
BRDT-BD2

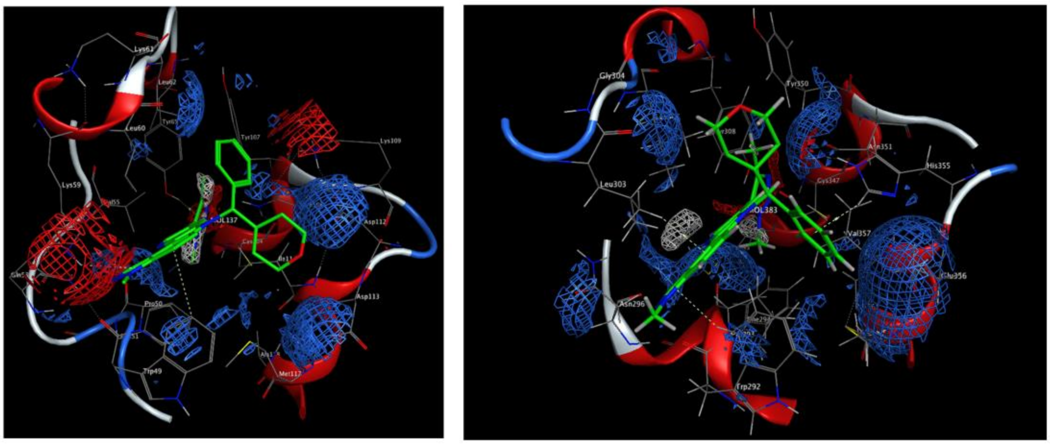

Supplementary Figure S13. The electrostatic map of the interaction site of BRDT and NHWD870 The electrostatic map near the interaction site of BRDT and NHWD870 after 50 ns molecular dynamics simulation which can provide the roadmap to further optimize the structure of NHWD870 to improve its binding to BRDT.

Supplementary Table S1. Sequence of primers used for RT-PCR in this study

Gene	Forward primer (5'→3')	Reverse primer (5'→3')
Actin	CACTGTCGAGTCGCGTCCA	CCACGATGGAGGGGAATACAG
Adam1b	GAATGGTGTGGTGGAGGACT	CCAGGTCACATGGTCCGTC
Akap4	CTGTGGTACTACAACGATGTCTG	AACTCGCCTTCTGACCTGGA
Anxa2	CAGCCTGGAGGGTGATCATT	TGGTGACCTCATCCACTCCT
Brd4	GACCACCTTCAGTCGTCCAG	TCACAGGCCTCCCTATGTCT
Brdt	AGGTGATTGCAAGCGAGTATTG	GATGCTTTCACTGGCTTCCC
Cabs1	ATGGCTGAAGATGGATCGCC	CGTGGTCTCCTTCCGAGGTA
Catspere	CGCCAAGGTGTCCTGATACT	ACAACCTTCCGGTATATCCCAC
Ccdc181	CTGCATCTTCCACAGATGGCT	TCATTCCTTCTGGGCAAGGC
Cd55b	ACAGAAGCTTCACCAACACCA	TGCAATAACAGCAACAAATCCATA
Cetn4	CGTTGGGTGATGGCCTCTAA	AGTTCCAGTTCCATCAGCATCA
Dlat	GACAAGGCCACCATAGGCTT	CCTGTAGTCTGCAAATGCTGC
Fbp1	CCAGTGTCGCATCTGACAGTTA	AGCCAGCGATACCATAGTTATAC
Gtpbp2	CACTTCTCTCTGTAGCTGGTGT	AGTTCAGACCTGGTTGAGTCC
H1-0	GCCAAAAAGCCCAAGGTTGT	GAAGGAGTGTCCCCAAGCAA
Hk1	GAGTCCCTGAAACACCTGGG	GCGGGGTTTTCTGATCCTTC
Hspa1l	CGGTTTGAAGAGCTGTGTGC	TTTTGCACCTTTGGGATGCG
Hspa2	AGGGAAGGCGAGAACCGAT	GGGCAGACATCCTGACGTT
Kcnq2	AGGTCTGATCCAGTCTGCCT	GGATGAGTCTGTACATGGGGAC
Kif2b	GTTTTGACCACGCCTTCGAC	GCCCATCGTGTGGGTTTTTC
Knstrn	GCATCAAAGCAGGAACCCAC	AGGCAAAGCATACCCTGTAGC
Ldhc	GCCATTGTAGGCCTGCTCAA	TAATAGCACACGCCATGCCC
Maats1	GTGACCATCCAGGAACCCAG	CGACACAATAAATAACGGGTCGTA
Nme5	GCTCTGGACCTCTTGTTGCTA	ATATCGCCCTTAAGCTGTCCG
Odf2	AAAACGAGGGAGTGTGGGTC	GCGTAGGCCAAGAAAGGGTT
Pdha2	CGTTGGAGGTTCCCATTCCA	CTTGGCTTTGTACGGCGATG
Pfkp	CGGGGACTCTGCGCTGTA	GGACAGGAGTGACCTGGTTT
Pick1	GAGACAGAAGGGTTGCCTGC	AATAGGAAACTGGGTCTGCCG
Pold2	CAAGGAAGCCAACGTCACATC	CTGAACCTTGTTCACTTGGGC
Pold3	ATTCGGGAGCTCAGCTGCAT	TGTATGGCACTAAACCTTCCAGT
Rad23a	CTAACGCCCCGCTATGTGG	CGCTAAGCTCGGGTACCATT
Rfc3	CTGCGCAACCTGACTCCATC	GGCCACTGTCTTCAGCATCT
Rfc4	CAGGTACTGGCAAGACAGCC	AGCCAAAATTACCTTTTGGATCTGT
Rimbp3	CTCGGAGATAACTCGGCACC	TGTGCCTCTGTCTCCTCGTA
Rsbn1	ACGCTGCGTACTATGCGTTA	GGATGCATTTCTGGGATAGACTGA
Rsph1	GAGGAGGGAGAGAACGACCT	TCAGCCCACTCCCCTTCATA
Spa17	CTCAAGCCTTCAGTACGCGA	TCTCAACTTGTTCTTGTTCCTCT
Spaca1	GCTGAGCTCTATGAGGTGCG	AATCGCATCCTGTGCCTACC
Sun3	TTGGCTTATGAGTCGCTTAAATG	CCACTGATGATTTAGAAGTCCTGAT
Tekt2	GTGAGGGGAAACCAGGCTC	GCCATGGCTAAGGAGACGAA
Txndc2	GAAAGGGGCAGCAACGAAAA	GTGTATTGCTGACCTTGGAAGC
Wdr35	GGATCTCTGTTAGCTAGGTACG	AAAGCAGAAGTGGCCTTATACA
Zpbp	CCTCGAAGTCAAGGGAGCC	GCCAAATGTCCAACTGGCG

Supplementary Table S2. The IC_{50} values of protein binding inhibition

Compound	Parameter	BRD2(D1,D2)	BRD3(D1,D2)	BRD4(D1,D2)	BRD4(D1)	BRD4(D2)	BRDT(D1)
NHWD870	Bottom	=0	18	12	7	16	19
	Top	101	99	109	101	96	101
	HillSlope	1.5	3.8	2.3	2.5	2.2	2.8
	IC_{50} (nM)	0.33	0.60	0.64	0.34	0.66	0.69
AZD5153	Bottom	-1	-16	-18	3	-7	3
	Top	100	101	=100	102	100	102
	HillSlope	0.9	1.0	0.6	1.0	1.0	1.3
	IC_{50} (nM)	1.3	5.1	0.66	35	49	81

Supplementary Table S3. The sample information of GSE39909 and NHWD870 of the interference RNA-seq

Genotype	Age	Sample Size
BRDT KO-/-	17 DPP	6
BRDT KO+/-	17 DPP	6
BRDT BD1-/-	20 DPP	6
BRDT BD1+/-	20 DPP	2
BRDT BD1+/+	20 DPP	4
BRDT KO-/-	20 DPP	6
BRDT KO+/+	20 DPP	6
NHWD870	9 weeks	3
CTL	9 weeks	3
